# Scientific knowledge graph and ontology generation using open large language models

**DOI:** 10.1039/d5dd00275c

**Published:** 2026-02-16

**Authors:** Alexandru Oarga, Matthew Hart, Andres M. Bran, Magdalena Lederbauer, Philippe Schwaller

**Affiliations:** a Laboratory of Artificial Chemical Intelligence (LIAC), ISIC, EPFL Switzerland philippe.schwaller@epfl.ch; b National Centre of Competence in Research (NCCR) Catalysis, EPFL Switzerland; c Department of Mathematics and Computer Science, University of Barcelona Spain; d Department Applied Physical Sciences, UNC Chapel Hill USA; e Department of Chemistry and Applied Biosciences, ETH Zurich Switzerland

## Abstract

Knowledge graphs (KGs) are powerful tools for structured information modeling, increasingly recognized for their potential to enhance the factuality and reasoning capabilities of Large Language Models (LLMs). However, in scientific domains, KG representation is often constrained by the absence of ontologies capable of modeling complex hierarchies and relationships inherent in the data. Moreover, the manual curation of KGs and ontologies from scientific literature remains a time-intensive task typically performed by domain experts. This work proposes a novel method leveraging LLMs for zero-shot, end-to-end ontology, and KG generation from scientific literature; implemented exclusively using open-source LLMs. We evaluate our approach by assessing its ability to reconstruct an existing KG and ontology of chemical elements and functional groups. Furthermore, we apply the method to the emerging field of Single Atom Catalysts (SACs), where information is scarce and unstructured. Our results demonstrate the effectiveness of our approach in automatically generating structured knowledge representations from complex scientific literature in areas where manual curation is challenging or time-consuming. The generated ontologies and KGs provide a foundation for improved information retrieval and reasoning in specialized fields, opening new avenues for LLM-assisted scientific research and knowledge management.

## Abstract 2

Knowledge graphs (KGs) are powerful tools for structured information modeling, increasingly recognized for their potential to enhance the factuality and reasoning capabilities of Large Language Models (LLMs).

## Graphical abstract

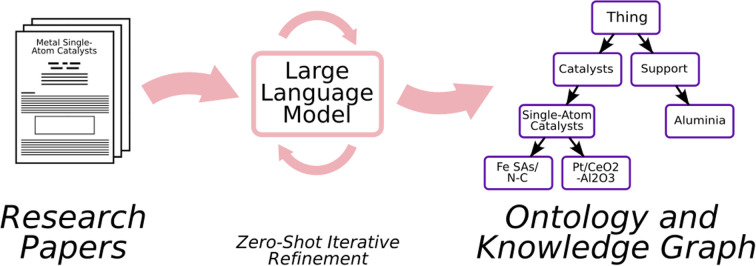

## 1 Introduction

Knowledge Graphs (KGs) are a powerful tool for representing structured information, enabling inference, retrieval, and analysis over large amounts of data.^1^ KGs model the real world in a graph representation, where nodes represent entities and edges represent relationships between them.^2^ KGs have shown use in the field of Natural Language Processing (NLP) by demonstrating an ability to enhance the capabilities of Large Language Models (LLMs).^3–5^ It is well-established that LLMs ‘hallucinate’ by, at times, generating factually incorrect or misleading information.^6^ In this context, KGs have been shown to mitigate this problem through Graph-Based Retrieval Augmented Generation (RAG), where graph structure is embedded as textual information as supplemental input to a LLM.^7–10^ Moreover, KGs have also been shown to improve the reasoning capabilities of LLMs.^11–13^

Knowledge Graph Extraction (KGE) from text is usually executed *via* the extraction of semantic triples of the form (subject, predicate, object) where subjects and objects are entities found using Named Entity Recognition (NER), and predicates (relationships between entities) are inferred from the context of the text.^14^ Currently, LLMs are state-of-the-art in many NLP tasks, including NER,^15,16^ making them promising candidates for unsupervised KGE.^17–19^ KGs based on triples alone, however, often fail to model scientific fields such as bioinformatics or chemistry where more complex structures are needed.^20–22^ Additionally, most KGE methods are pre-trained on specific text corpora or domains or require labelled subsets of samples, which hampers their ability to generalize to different contexts.^23,24^ These factors limit the applicability of KGE to niche domains in scientific literature.^25^

In complex domains, the enrichment of KGE with ontologies can significantly improve information representation and successful semantic triple extraction. Ontologies are formalized representations of knowledge consisting of individuals, classes, relations, and attributes. In the context of a KG, these serve as the structure of allowed relationships between entities, and provide a consistent, standardized vocabulary for the data in the graph. Thus, ontologies provide a high-level schema for the knowledge present in the KG.^2,26^ Domain-specific ontologies however require a construction process that is typically time-consuming, labour-intensive, and outside of the scope of skills of domain scientists, often limiting their presence in several specialized subjects.^27,28^

Concurrent with the success of LLMs in similar tasks, some solutions have been proposed for ontology generation using LLMs.^29,30^ These methods, however, often have limitations: they may require pre-defined training sets for fine-tuning,^31–33^ cannot generate full ontologies,^34^ do not scale beyond smaller problems,^35,36^ or solely rely on the LLMs knowledge, making their application to scientific fields unpractical or unclear for generalisation into other domains.^37,38^ There is a clear need to generate hallucination free knowledge extraction from scientific text in a way that is human-interpretable, consistent, and does not require efforts in fine-tuning language models on niche subjects.^39^

The contributions of these paper are threefold: (1) we propose a zero-shot pipeline for vocabulary and taxonomy extraction directly from scientific literature using LLMs, leveraging an ontological knowledge schema and minimal manual intervention, (2) we evaluate the proposed method's ability to reconstruct an existing knowledge graph (KG) and chemical element knowledge schema, demonstrating that it outperforms existing methods from the literature, and (3) we apply the method to the scientific domain of Single Atom Catalysis (SAC),^40–43^ an emerging field in catalysis with no previously existing KG. In doing so, we create what is, to our knowledge, the first example of a domain-specific ontological schema and knowledge graph for SAC, and show that the combination of an extracted knowledge schema and a KG improves the quality of RAG-based solutions that rely solely on KGs, such as GraphRAG.^10^ We demonstrate the value that general-purpose LLMs add to automating the typically labour-intensive process of ontology construction. The presented work therefore, represents an advancement in the use of LLMs to perform domain-specific knowledge extraction in a way that can be interpreted by experts.

## 2 Methods

### 2.1 Ontologies as a form of knowledge representation

From a practical engineering perspective, ontologies are a specification of conceptualization using description logic. They model relationships between concepts and the words used to represent them. The so called “backbone” of an ontology can be thought of as a series of “isA” relationships formed in a hierarchical tree structure called a taxonomy.^44^ This can be further engineered to include relationships, classes, instances, properties, rules, and axioms.^45^ For the purpose of this work, we use the following, approximate, definition of an ontology:

**Definition 2.1** *We take an ontology to be a conceptualization* {*C,R,I*}*where:*

*• C is the set of all classes present in the ontology C =* {*c*_*1*_*,c*_*2*_*,c*_*3*_*,…,c*_*n*_}*, such that* ∀*c* ∈*C is instantiable.*

*• R is the set of relation types in the ontology, R* = {*r*_*1*_*,r*_*2*_*,…,r*_*m*_}*, and T is the set of relation instances (triples) such that T* = {(*c*_*i*_*,r,c*_*j*_)|*c*_*i*_*,c*_*j*_ ∈*C and r* ∈ *R*}

*• I is the set of all instances of concepts present in the ontology, I* = {*i*_*1*_*,i*_*2*_*,…,i*_*n*_}*such that ∀i ∈ I,*∃*c* ∈ *C*: *c(i)*

This definition represents a computational approximation to formal ontological frameworks rather than a complete logical ontology. This design choice reflects the practical constraints of automated extraction from natural language text using current LLM technology. Our focus is on capturing the essential structural and hierarchical relationships that enable improved knowledge organization and retrieval, rather than full logical reasoning capabilities. Noteworthy is that our approach does not employ formal upper-level ontologies that provide foundational ontological commitments.^46^ Instead, we extract domain-specific categories directly from the literature, which results in a more practical but less formally grounded taxonomic structure.

Seeing that a key practical application of ontologies is the construction and integration of databases.^27^ We demonstrate the usefulness of our pipeline by using constructed ontologies to create knowledge graphs. To relate knowledge graphs to ontologies, we utilize the following definition:

**Definition 2.2** *A knowledge graph is set of tuples* {*S,P,O*}*where*

• *S is the set of subjects where s* ∈ *S is an instance of a concept from the ontology* ⇒ *S* ∈ *I*

• *P is the set of predicates where p* ∈ *P is a relation from the ontology* ⇒*p* ∈ *R*_∫_

• *O is the set of objects where o* ∈ *O is also an instance of a p concept from the ontology such that p*: 
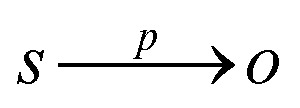


From this definition, it follows that the ontology serves as the conceptual schema for the construction of a knowledge graph.^47^ The following methods detail our approach to leveraging zeroshot learning for creating an ontology of SAC and the subsequent construction of a consistent knowledge graph.

**Fig. 1:**
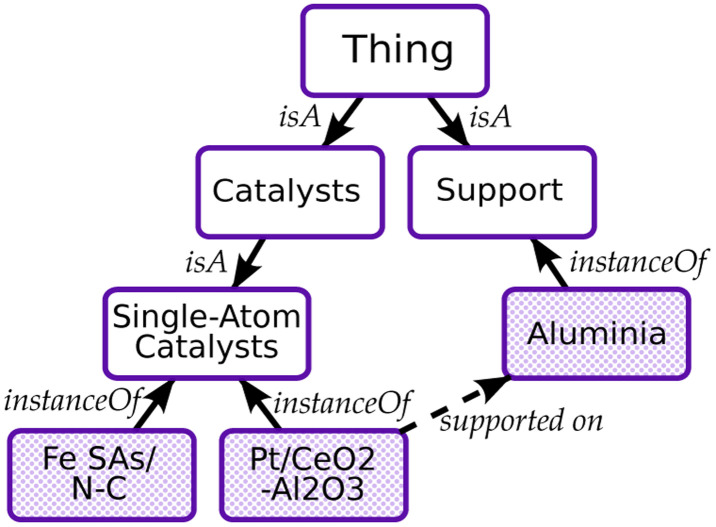
Illustrative example of an ontology and a knowledge graph in the domain of SAC.

**Example 2.3** Fig. 1 shows an example ontology schema with four classes (white nodes) and a KG with three instances (shaded nodes). The ontology has three isA and is related to the KG through three instance of relationships. Between the nodes of the KG, one relationship exists of the type ‘supported on’. The term ‘Thing’ is always the root node of an ontology.

### 2.2 In-context ontology generation

**Fig. 2:**
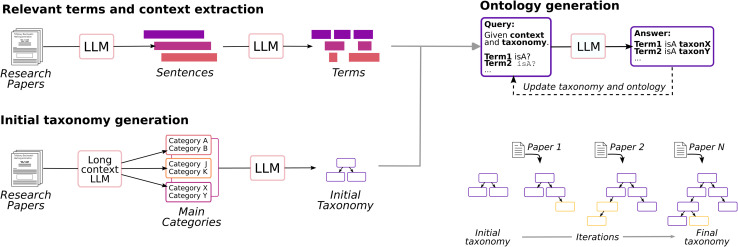
Illustration of the iterative and incremental taxonomy generation process.

Here, we introduce OntoGen, our method for in-context ontology generation and KGE from scientific literature. The method is split into five separate steps: (1) the vocabulary that will be used to generate the ontology is extracted from the text, (2) the initial categories of the ontology are generated, (3) the taxonomy of the ontology is extracted, (4) the knowledge graph is instantiated from the extracted ontology, and (5) the relationships between the elements of the vocabulary are extracted (Fig. 2).

#### 2.2.1 Vocabulary extraction

The first step in our method is the extraction of all the domain specific vocabulary terms from a given set of text. The extracted words serve as the base vocabulary that will compose the knowledge representation schema. It follows that any terms that are not extracted in this step will be absent from the final vocabulary. Vocabulary extraction is itself split into three steps: (1) terms extraction, (2) acronyms extraction, and (3) lemmatization.

##### 2.2.1.1 Terms extraction

Traditional models trained for specific NER tasks often fail to generalize to domains not included in their training data.^48^ To address this limitation, we utilize open general purpose LLMs for zero-shot vocabulary identification. The relevant text is first split into sentences to ensure individual words can be obtained in a fine-grained, detailed manner. This sentence segmentation is performed using a pre-trained LLM that recognizes sentence boundaries. Empirically, it was observed that splitting the text into smaller sentences allowed for a more fine-grained extraction and increased the number of terms extracted. For each of the extracted sentences, an LLM is then prompted to list each of the terms present in the sentence, which acts as the set of vocabulary extracted from the text. Finally, to avoid terms hallucinated by the LLM or subtle changes in the writing of the terms, a verification step is automatically performed to discard those terms not present in the original text.

##### 2.2.1.2 Acronym extraction

Acronyms are commonly used in scientific literature to shorten longer or redundant vocabulary.^49^ To be able to associate each of the extracted terms of the previous step with their respective acronyms, we employ a separate procedure similar to terms extraction, but with the LLM prompted specifically to list all acronyms and their corresponding terms. As in the previous step, a verification procedure is used to ensure that the pairs of acronym terms listed are present in the original text.

##### 2.2.1.3 Lemmatization

To handle different writing forms of the same term (*e.g.* “Single-Atom Catalyst” and “Single Atom Catalysts”), we apply lemmatization to each extracted term after removing punctuation. This process reduces words to their root form, avoiding grammatical variations and synonyms (*e.g.* “Single-Atom Catalysts” becomes “Single Atom Catalyst”). Terms that are mapped to the same root form after lemmatization are considered to refer to the same concept. This is particularly useful in the taxonomy generation process, where the same concept can appear in different forms across different papers. When such terms are identified, they are merged into a single term in the taxonomy.

#### 2.2.2 Category extraction

For the first level of the taxonomic hierarchy, we include the main classes that the representation schema will cover. For instance, in the field of chemistry, this might include elements, compounds, and reactions. However, these high-level categories cannot always be extracted from individual papers, as each scientific paper typically focuses on a particular subdomain. Therefore, the highest level classes must be extracted across several of the papers available to the system.

To accomplish this, we propose a two-step process:

(1) Generation: generating a list of categories from a set of papers is challenging for conventional LLMs due to their limited context window. To overcome this limitation, we employ Long-Context LLMs (LCLLMs)^50–53^ for this task. First, a random sample of papers is presented to an LCLLM, which is then prompted to extract the main categories across the given papers. As LCLLMs are known to be sensitive to input order,^54^ we generate multiple answers, each with a random shuffle in the order of the papers.

(2) Refinement: using the answers generated in the previous step, we prompt an LLM to generate a curated list of categories using the most frequently occurring ones. To obtain categorial consistency, we apply self-consistency in this step. We run the same prompt multiple times and select the majority vote among the answers. The formal definition of self-consistency is recalled in the SI.

It is important to note that the outcome of the previous step is a list of categories that will serve as the seed for the rest of the representation schema. However, the usefulness of the ontological framework (from a practical perspective) depends on the downstream application that it is intended for. Therefore, some manual curation effort may be required at the end of this step to select the most relevant categories according to the specific goals of the user.

#### 2.2.3 Taxonomy extraction

The relationships in our extracted taxonomic structures consist of *isA* relationships in the form of {(*c*_*i*_,*r*,*c*_*j*_)|*c*_*i*_,*c*_*j*_ ∈ *C* and *r* = *isA*}. Once the vocabulary is extracted and the main categories are generated, the vocabulary needs to be organized into a hierarchical structure. Since the first level of the hierarchy has already been established, a top-down approach^27^ is the most logical method for generating the taxonomy. Although taxonomic information can be extracted from each paper separately, it must ultimately be consolidated into a global unified taxonomy. Therefore, an incremental and sequential approach is proposed for constructing a global taxonomy.

Let *P* = {*P*_1_,*P*_2_,…,*P*_*N*_} be a corpus of *N* papers, *V* = {*V*_1_,*V*_2_,…,*V*_*N*_} a the set of terms extracted from the papers, where *V*_*i*_ is the vocabulary from paper *P*_*i*_, and *T*^(*k*)^ = {(*s*_1_,*t*_1_),(*s*_2_,*t*_2_),…(*s*_*M*_,*t*_*M*_)} a taxonomy at iteration *k*, where each (*s*_*i*_,*t*_*i*_) with *s*_*i*_,*t*_*i*_ ∈ *V* is a pair of terms that represents a *isA* relationship. The procedure to generate the full taxonomy is presented in Algorithm 1.
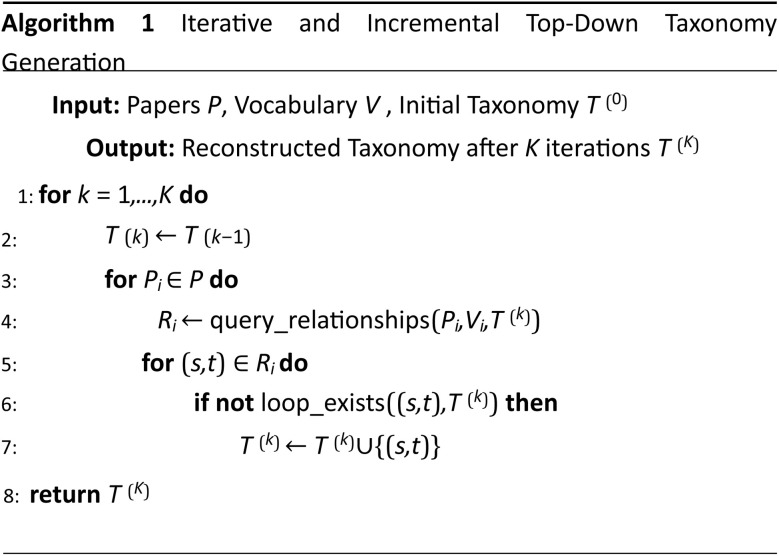


In this algorithm, query_relationships extracts pairs (*s*,*t*) of *isA* relationships based on the information presented in paper *P*_*i*_, where *s* is a term present in the taxonomy *T*^(*k*)^ and *t* is a term from the vocabulary *V*_*i*_ which will either represent a class or an instance in the ontological schema. The primary goal of query_relationships is to place each term into the existing taxonomy. Note that query_relationships may not necessarily return a single relationship for each term, but rather a subset of relationships. Since we want to generate a directed acyclic taxonomy, the loop_exists function checks whether a given relationship creates a loop in the taxonomy. If a loop is created, then the given relationship is discarded. This procedure builds a taxonomy incrementally, requiring multiple iterations since a term may not be placed in the earlier taxonomies but may find its position in a later one.

In our implementation, query_relationships prompts an LLM with the content of the paper, the complete list of terms in the taxonomy, and the vocabulary terms to be queried. An example of such a prompt and its corresponding answer can be found in the SI. Given that the quality of the generated taxonomy heavily depends on the performance of this specific function, self-consistency is employed to mitigate the number of hallucinations generated, this is, for each query, many samples are generated and the majority voting of the answers is taken as final answer.

#### 2.2.4 Knowledge graph instantiation

Once the full taxonomy is constructed, the leaf nodes of the taxonomy are considered to be instances for the KG. We reason that since leaf nodes have no children, they should be treated as the lowest-level concepts in our knowledge representation schema, and thus are assumed to be instances of the KG. Conversely, nonleaf nodes, having children related through *isA* relationships, are considered classes in the ontological format rather than instances for the KG.

#### 2.2.5 Relationship extraction

Finally, we perform relationship extraction using a conventional triplet extraction approach. Specifically, we prompt an LLM to generate triplets from a given text, considering the extracted vocabulary. The resulting triplets are then filtered to retain only those involving terms from the vocabulary.

**Fig. 3:**
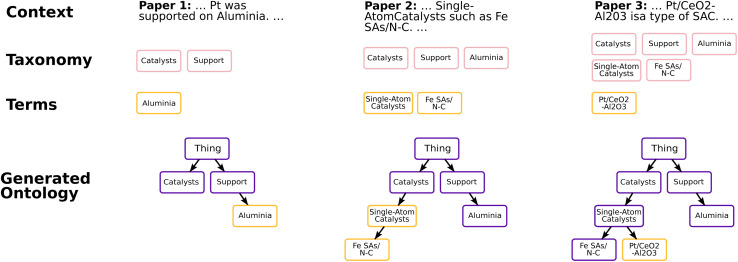
Illustration of the iterative and incremental taxonomy generation process.

The overall complexity of the method is reported in Table 1, where *N* is the number of papers, *S*_G_ and *S*_R_ are the number of samples in both steps of categories extraction, *K* is the number of iterations, *L* is the length of a paper and *T* is the size of a taxonomy. Notice that, except for the categories extraction step, only one paper at a time is processed in each LLM call (Fig. 3).

**Table 1:** Computational complexity of each step in in-context ontology generation and KGE in terms of LLM calls and prompt length

Step	LLMs calls	Prompt length
Vocabulary extraction	*O*(*N*)	*O*(*L*)
Categories extraction	*O*(*S*_G_ + *S*_R_)	*O*(*L*·*N*)
Taxonomy extraction	*O*(*K*·*N*)	*O*(*L* + |*T*|)
Relationships extraction	*O*(*N*)	*O*(*L*)

Details on the packages and models used for the implementation of the pipeline are found in the SI.

### 2.3 Ontology reconstruction evaluation

Evaluating an engineered ontology is widely considered nontrivial,^55^ as the quality of the ontology is highly dependent on the downstream application for which it is intended. Additionally, many valid ontology schemas can be generated from the same text. To overcome the limitations of common metrics such as term accuracy, the percentage of the terms in the ground truth taxonomy that appear in the reconstructed taxonomy, and knowledge graph accuracy, the percentage of the instances in the ground truth KG that are also instances of the reconstructed KG, we propose the hierarchical accuracy as an evaluation metric for reconstructed ontologies.

#### 2.3.1 Taxonomy evaluation

The evaluation of taxonomy reconstruction is a subtle and far from trivial problem.^56,57^ A reliable evaluation requires assigning partial credit to reconstructed taxonomies that capture structural similarities. For example, a method should receive credit for correctly placing nodes within the right subtree, or not receive penalization for including additional information (not necessarily incorrect) not present in the ground truth taxonomy. In this work, we propose evaluating taxonomy reconstruction using a combination of complementary metrics, each of which highlights different aspects of performance. Particularly, we adapt the notions of hierarchical precision, recall, F1-score, and accuracy, originally proposed to evaluate tree-like structures.^56^ However, in our case, a node may belong to multiple subtrees, meaning the taxonomy is not strictly a tree but a directed acyclic graph (DAG). We therefore generalize these metrics to handle DAG structures where nodes may have multiple parents.

We now introduce the metrics formally. First, we treat a taxonomy as a triplet *G* = (*N*,*E*), where *N* is the set of nodes and *E* is the set of directed edges. The graph has a unique root node *r*, such that at least one directed path exists from *r* to every node *n*_*i*_ ∈ *N*. Formally, for any *n*_*i*_ ∈ *N*, a path from root to node is defined as:

1 path(*n*_*i*_) = (*n*_0_,*n*_1_,…,*n*_*i*_)∣*n*_0_ = *r*,(*n*_*j*_,*n*_*j*+1_) ∈ *E*.

Let us also denote *P*_*n*_*i*__ to be the set of all paths from *r* to node *n*_*i*_. Let *G̃* = (*Ñ*,*Ẽ*) be the ground truth taxonomy. Since multiple paths may correspond between the reconstructed taxonomy and the ground truth, we define a similarity function to match paths. For a ground truth path:2
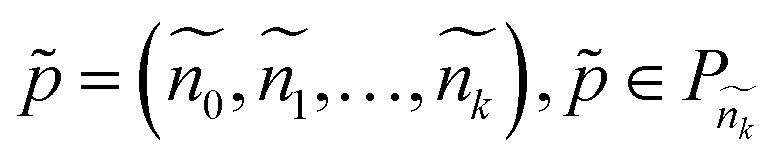
the most similar reconstructed path *p* ∈ *P*_*n*_*k*__, ending in the same node (*i.e.* semantically nodes *n*_*i*_ ∈ *N* and *ñ*_*i*_ ∈ *Ñ* are the same), is defined as:3
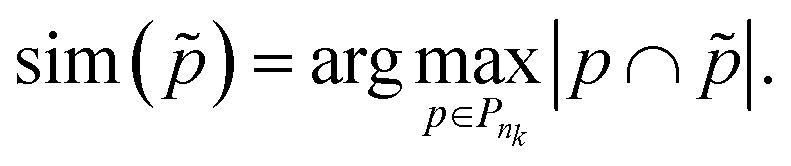


We can now define the proposed metrics. We define hierarchical recall (hR) and hierarchical precision (hP) as follows:4
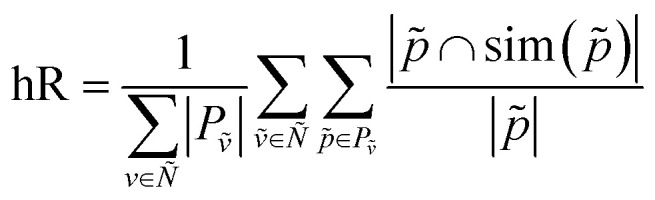
5
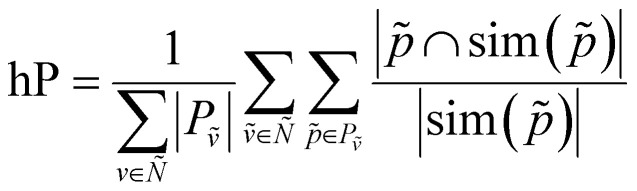


We can then generalize hierarchical accuracy (hAcc) as the average number of paths with at least one common ancestor:6
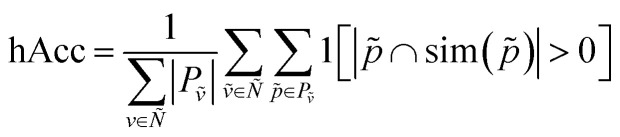


Beyond these general metrics, we also employ well-established measures for taxonomy evaluation, including: Lowest Common Ancestor (LCA), where for each pair of nodes in the ground truth, we check whether their lowest common ancestor is preserved in the reconstructed taxonomy, and Ancestor Error Count (AEC), where for each ground truth path, we compute the average number of discrepancies between the path and its most similar reconstructed path.

## 3 Results & discussion

Here we present the results obtained with the proposed method for ontological knowledge representation and knowledge graph generation from scientific literature. The performance of the method is first evaluated by measuring the ability to reconstruct an existing KG and ontology of chemical elements and functional groups from a corpus of text. The method is then applied to construct the first KG and ontology of the scientific field of SAC. The effectiveness of the ontology is then evaluated by applying it in a RAG-based solution for SACs domain.

### 3.1 Chemical elements and functional groups reconstruction

ElementKG^58^ is an ontology and KG of chemical elements and functional groups with 272 nodes, of which 188 are instances. This ontology and KG were curated manually by domain experts from information primarily sourced from Wikipedia. To reconstruct ElementKG, 76 Wikipedia pages corresponding to functional groups (*e.g.* alkyne, alkane, *etc.*) and groups of elements (*e.g.* noble gases, metalloids, *etc.*) were collected for our work. Except for the functional groups page, only the introduction section of the pages was used for the reconstruction, as it was observed to contain most of the relevant taxonomical information. Additionally, four plain texts were manually curated for cases where no Wikipedia page exists (*e.g.* ferrous elements) or the information available is not enough to reconstruct ElementKG (*e.g.* rare metals).

Table 2 reports the quantitative results, showing how the considered taxonomy and knowledge graph metrics evolve over successive iterations of our algorithm. The table also reports the metrics for each of the two main subcategories in ElementKG, that is, elements and functional groups. We can see that our method achieves high accuracy in reconstructing the ElementKG taxonomy. In the final iteration, both term and hierarchical accuracy exceed 90%, the hierarchical F1 score is above 0.73, and leaf node accuracy approaches 75%, indicating a good performance in reconstruction. Notably, most metrics improve steadily over iterations. For instance, overall term accuracy rises from 80.2% to 92.8%, while hierarchical F1 improves from 0.64 to 0.73. These results highlight the effectiveness of our incremental approach, where new terms are correctly placed in the taxonomy over time, refining the structure iteratively. In the SI, we include qualitative results where we include failure cases of the ElementKG reconstruction. There, we can see that one limitation in the reconstruction is the absence of intermediate terms such as ‘Groups Containing Sulfur’ in the functional group reconstructed subtree.

**Table 2:** Evaluation metrics for the reconstruction of ElementKG averaged over five runs. We observe that the quality of the reconstruction improves with increasing number of iterations

Category	Metric	Iteration 0	Iteration 1	Iteration 2	Iteration 3
All	Term accuracy (↑)	0.802 ± 0.078	0.900 ± 0.032	0.919 ± 0.020	0.928 ± 0.014
	Hierarchical precision (↑)	0.775 ± 0.077	0.834 ± 0.059	0.842 ± 0.048	0.836 ± 0.038
	Hierarchical recall (↑)	0.556 ± 0.045	0.627 ± 0.029	0.638 ± 0.026	0.660 ± 0.024
	Hierarchical F1 (↑)	0.646 ± 0.047	0.715 ± 0.027	0.725 ± 0.015	0.737 ± 0.023
	Hierarchical accuracy (↑)	0.825 ± 0.061	0.902 ± 0.025	0.917 ± 0.015	0.924 ± 0.011
	Ancestor error count (↓)	2.865 ± 0.197	2.687 ± 0.132	2.673 ± 0.142	2.586 ± 0.192
	Lowest common ancestor (↑)	0.459 ± 0.105	0.576 ± 0.060	0.594 ± 0.042	0.621 ± 0.049
	Leaf nodes accuracy (↑)	0.716 ± 0.076	0.759 ± 0.044	0.775 ± 0.020	0.780 ± 0.020
Element	Term accuracy (↑)	0.906 ± 0.022	0.949 ± 0.029	0.959 ± 0.032	0.961 ± 0.029
	Hierarchical precision (↑)	0.845 ± 0.058	0.855 ± 0.058	0.859 ± 0.061	0.839 ± 0.057
	Hierarchical recall (↑)	0.524 ± 0.077	0.573 ± 0.057	0.578 ± 0.053	0.613 ± 0.056
	Hierarchical F1 (↑)	0.640 ± 0.045	0.682 ± 0.035	0.688 ± 0.032	0.707 ± 0.049
	Hierarchical accuracy (↑)	0.916 ± 0.014	0.944 ± 0.018	0.950 ± 0.020	0.951 ± 0.018
	Ancestor error count (↓)	3.707 ± 0.336	3.510 ± 0.232	3.504 ± 0.234	3.372 ± 0.343
	Lowest common ancestor (↑)	0.251 ± 0.050	0.291 ± 0.060	0.293 ± 0.061	0.341 ± 0.091
	Leaf nodes accuracy (↑)	0.747 ± 0.000	0.747 ± 0.000	0.747 ± 0.000	0.747 ± 0.000
Functional group	Term accuracy (↑)	0.697 ± 0.156	0.850 ± 0.067	0.878 ± 0.019	0.894 ± 0.010
	Hierarchical precision (↑)	0.624 ± 0.212	0.747 ± 0.180	0.760 ± 0.146	0.765 ± 0.132
	Hierarchical recall (↑)	0.440 ± 0.077	0.541 ± 0.048	0.557 ± 0.031	0.569 ± 0.029
	Hierarchical F1 (↑)	0.511 ± 0.128	0.621 ± 0.100	0.636 ± 0.071	0.647 ± 0.061
	Hierarchical accuracy (↑)	0.691 ± 0.156	0.844 ± 0.066	0.872 ± 0.018	0.888 ± 0.010
	Ancestor error count (↓)	1.580 ± 0.116	1.423 ± 0.114	1.398 ± 0.082	1.375 ± 0.076
	Lowest common ancestor (↑)	0.372 ± 0.231	0.530 ± 0.216	0.534 ± 0.213	0.547 ± 0.210
	Leaf nodes accuracy (↑)	0.675 ± 0.177	0.775 ± 0.103	0.812 ± 0.046	0.825 ± 0.046

**Fig. 4:**
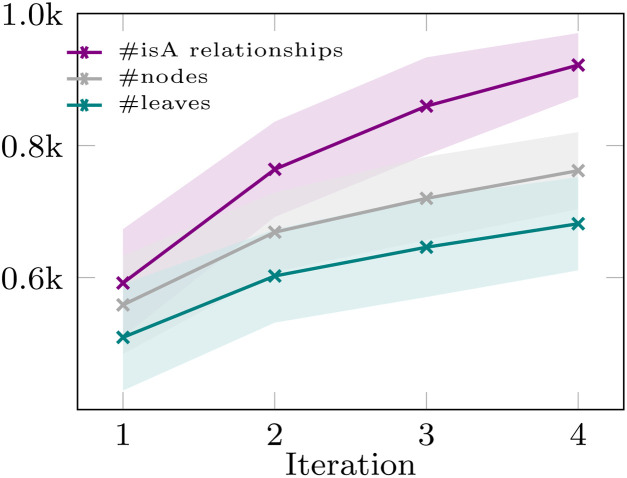
Mean number of *isA* relationships, nodes and leaves (blue lines) and standard deviation (shaded areas) averaged over two runs.

Fig. 4 provides descriptive statistics of the reconstructed taxonomy. The figures illustrate how the number of *isA* relationships increases constantly over iterations. We observed that the root cause of this trend is the inclusion of broad, general terms (*e.g.* Materials) that are placed in the taxonomy, which naturally accumulate many descendants. Further statistics related to the reconstruction can be found in the SI.

To evaluate our approach we compare against existing methods in the literature for extracting ontology information from text using LLMs. Particular we considered Large Language Models for Ontology Learning (LLM4OL),^25^ which queries each pair of terms separately to extract hierarchical relationships, and Ontology Learning from Text (OLfT),^59^ which extracts all relationships from a given text and then aggregates them into the final taxonomy.

Finally, Table 3 compares our method against baseline LLMbased ontology generation approaches. In this table, we can see that our approach outperforms all baselines across nearly all metrics considered. For instance, overall our methods achieve 92.8% term accuracy, compared with 43.8% from OLfT and 87.4% from LLM4OL. For hierarchical reconstruction, we achieve a hierarchical F1 of 0.73 compared with 0.33 from OLfT and 0.64 with LLM4OL. Regarding leaf node accuracy, our method reaches 74.9% outperforming 33.6% from OLfT and 72.1% from LLM4OL.

**Table 3:** Baseline comparison for the reconstruction of ElementKG averaged over five runs. Our approach generally outperforms the baselines in the metrics considered. Best values are highlighted in bold

Category	Metric	OLfT []	LLM4OL []	OntoGen (ours)
All	Term accuracy (↑)	0.438 ± 0.042	0.874 ± 0.010	**0.928 ± 0.015**
	Hierarchical precision (↑)	0.388 ± 0.034	0.831 ± 0.066	**0.837 ± 0.038**
	Hierarchical recall (↑)	0.298 ± 0.041	0.534 ± 0.005	**0.660 ± 0.025**
	Hierarchical F1 (↑)	0.337 ± 0.039	0.649 ± 0.018	**0.737 ± 0.023**
	Hierarchical accuracy (↑)	0.430 ± 0.071	0.880 ± 0.003	**0.924 ± 0.012**
	Ancestor error count (↓)	3.349 ± 0.068	3.227 ± 0.157	**2.587 ± 0.193**
	Lowest common ancestor (↑)	0.112 ± 0.024	0.372 ± 0.173	**0.621 ± 0.049**
	Leaf nodes accuracy (↑)	0.336 ± 0.045	0.721 ± 0.010	**0.749±0.025**
Element	Term accuracy (↑)	0.438 ± 0.042	0.949 ± 0.003	**0.961 ± 0.029**
	Hierarchical precision (↑)	0.450 ± 0.052	**0.948 ± 0.001**	0.840 ± 0.058
	Hierarchical recall (↑)	0.313 ± 0.068	0.433 ± 0.001	**0.613±0.056**
	Hierarchical F1 (↑)	0.368 ± 0.064	0.595 ± 0.001	**0.707 ± 0.049**
	Hierarchical accuracy (↑)	0.544 ± 0.124	0.944 ± 0.002	**0.952 ± 0.019**
	Ancestor error count (↓)	4.173 ± 0.112	4.108 ± 0.002	**3.373 ± 0.343**
	Lowest common ancestor (↑)	0.090 ± 0.033	0.212 ± 0.000	**0.342 ± 0.091**
	Leaf nodes accuracy (↑)	0.393 ± 0.099	0.705 ± 0.014	**0.748 ± 0.000**
Functional group	Term accuracy (↑)	0.579 ± 0.107	0.793 ± 0.007	0.895 ± 0.011
	Hierarchical precision (↑)	0.233 ± 0.019	**0.783 ± 0.005**	0.766 ± 0.132
	Hierarchical recall (↑)	0.175 ± 0.014	0.473 ± 0.005	**0.569 ± 0.030**
	Hierarchical F1 (↑)	0.200 ± 0.016	0.589 ± 0.004	**0.647 ± 0.061**
	Hierarchical accuracy (↑)	0.257 ± 0.020	0.787 ± 0.007	**0.888 ± 0.011**
	Ancestor error count (↓)	2.065 ± 0.041	1.554 ± 0.004	**1.375 ± 0.076**
	Lowest common ancestor (↑)	0.052 ± 0.008	0.497 ± 0.009	**0.548 ± 0.211**
	Leaf nodes accuracy (↑)	0.223 ± 0.017	0.743 ± 0.037	**0.750 ± 0.058**

In the SI, we ablate the use of self consistency in our proposed method. We show that, without self-consistency, the method slightly improves overall hierarchical recall from 0.66 to 0.67. However, when self-consistency is applied, hierarchical precision increases from 0.74 to 0.83, and the hierarchical F1 score rises from 0.70 to 0.73. This means that removing self-consistency helps include more terms in the taxonomy; however, some of these newly introduced terms are not necessarily correct, which reduces the quality of the reconstruction. These results demonstrate that self-consistency enhances the quality of the generated taxonomy by mitigating noise from the LLM.

### 3.2 Ontology and KG generation for single atom catalysis

SAC, a recent scientific domain studying the catalytic properties of isolated single metal atoms,^40,42^ presents an ideal case study for our method due to its mostly unstructured literature. We applied our procedure to generate the first ontology and KG of this field, using a corpus of 20 papers. This corpus of SACs research papers, used for ontology generation, includes publications from top journals in catalysis and materials science from the past five years. The papers were carefully selected to cover various aspects of SACs, including synthesis methods, characterization techniques, and applications. The research papers were obtained from Wiley Journals through Wiley's official API.^60^

**Fig. 5:**
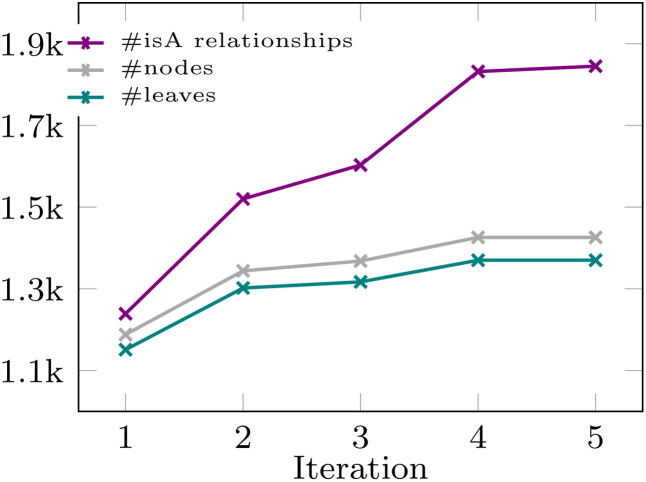
Number of nodes, *isA* relationships and leaves of the generated SACs taxonomy over iterations.

Focusing on the abstract and introduction sections, where concepts are typically introduced and explained, we extracted 1944 terms with 296 relationships. The category identification process initially yielded 331 categories, refined to 131, and manually curated to 12 key categories. In the SI, we include the whole list of 131 categories and how these were clustered by topic to create 12 key categories. Fig. 5 shows the evolution of the number of nodes, *isA* relationships and leaves over iterations. Further details are provided in the SI.

After five iterations, the growth in nodes, *isA* relationships, and leaves slowed significantly, possibly indicating convergence in taxonomy generation, despite not exhausting the entire vocabulary. This suggests the method's effectiveness in structuring knowledge from an emerging scientific field.

#### 3.2.1 Vocabulary extraction

First, we evaluate our proposed pipeline for term extraction. To do this, we manually extracted all terms from the abstracts of the 20 papers. For baseline comparisons, we consider two common approaches to term extraction: (i) a transformer-based method (*KeyBERT*^61^), and (ii) a hybrid approach, where spaCy^62^ is used to extract candidate terms followed by the statistical C/NC method^63^ to identify terms. For evaluation, we compute exact word matches, as well as partial matches based on substrings and single-word matches.

In Table 4 we present the quantitative comparison with the baseline methods. In this table, we show that our methods achieve an exact-match F1-score of 0.55, outperforming keyBERT (0.12) and C/NC (0.36). If we consider word-level matches, C/NC achieves higher recall (0.80) compared to our method (0.68). However, our method exceeds considerably in precision (0.94 compared to 0.65 for C/NC). As a result, our method achieves a higher overall F1 score (0.79 compared to 0.71 for C/NC). Examining the number of predicted words, we observe that our approach is more conservative, while both C/NC and KeyBERT tend to overpredict terms.

**Table 4:** Baseline comparison for vocabulary extraction in the SACs setting averaged over five runs. We report the metrics at different levels, including: exact term matching, substring matching and word-level matching. Our approach generally outperforms the baselines considered

	KeyBERT^61^ C/NC^63^	Ours
**Exact match**		
Precision (↑)	0.102 ± 0.035 0.333 ± 0.096	0.671 ± 0.150
Recall (↑)	0.172 ± 0.060 0.406 ± 0.090	0.483 ± 0.092
F1 (↑)	0.126 ± 0.042 0.363 ± 0.088	0.558 ± 0.112
# Gold terms	43.0 ± 15.4 43.0 ± 15.4	43.0 ± 15.4
# Predictions	71.8 ± 19.1 51.8 ± 11.0	31.6 ± 11.9

**Substring**		
Precision (↑)	0.335 ± 0.079 0.671 ± 0.077	0.909 ± 0.098
Recall (↑)	0.569 ± 0.130 0.836 ± 0.082	0.667 ± 0.083
F1 (↑)	0.416 ± 0.082 0.738 ± 0.043	0.762 ± 0.064
# Gold terms	43.0 ± 15.4 43.0 ± 15.4	43.0 ± 15.4
# Predictions	71.8 ± 19.1 51.8 ± 11.0	31.6 ± 11.9

**Word**		
Precision (↑)	0.329 ± 0.040 0.658 ± 0.066	0.947 ± 0.058
Recall (↑)	0.637 ± 0.123 0.802 ± 0.094	0.686 ± 0.103
F1 (↑)	0.429 ± 0.044 0.718 ± 0.048	0.790 ± 0.075
# Gold terms	94.7 ± 25.9 94.7 ± 25.9	94.7 ± 25.9
# Predictions	182.1 ± 50.3113.0 ± 22.0	68.0 ± 18.4

In the SI, we ablate both sentence splitting and verification of our vocabulary extraction pipeline. We show that without splitting text into sentences, the exact-match F1 score decreases from 0.55 to 0.52; without verification, it drops to 0.50.

Similarly, for word-level matches, the F1 score falls from 0.79 to 0.75 without sentence splitting, and from 0.79 to 0.77 without verification.

#### 3.2.2 GraphRAG-based downstream evaluation

To demonstrate the practical value of our generated ontology, we conducted a comparative study in the SAC domain. We evaluated two approaches: (1) a standard knowledge graph (denoted only KG), where the graph consists solely of extracted term-to-term triplets (excluding isA relationships), and (2) an enhanced approach (denoted KG + ontology), where the graph combines the knowledge graph with the generated taxonomy, *i.e.*, we extend the KG by adding isA relationships. The only difference between (1) and (2) is that the second approach incorporates isA relationships.^10^

The GraphRAG pipeline is composed of the following steps: (1) the generation of the KG from plain text, (2) description generation for each term and relationship, (3) clustering for community generation, (4) community description generation, and (5) community retrieval for answer generation. In our enhanced approach, we extended the KG in step (1) with our generated ontology (KG + ontology).

To evaluate performance, we used the same four metrics as the original GraphRAG study:^10^ comprehensiveness, diversity, empowerment, and directness. We created a dataset of 50 questions using long-context LLMs, covering various SAC topics such as synthesis, characterization methods, and applications, which were then validated by domain experts.

Following the GraphRAG methodology,^10^ we used an LLM, in our work Anthropic's Claude 3.5 Sonnet, to evaluate the quality of generated answers for both approaches. Table 5 presents the results, clearly showing that our KG + ontology approach significantly outperforms the standard KG-only method across all metrics. Additionally, an inter-rater reliability, measured using Cohen's Kappa, was obtained of *k* = 0.52 between human evaluation and LLM evaluation, indicating a moderate agreement between the two. This improvement demonstrates the ontology's ability to enhance term relationships, enabling better clustering and retrieval, ultimately resulting in more informative and higher-quality answers.

**Table 5:** Win-rate according to different criteria in the GraphRAG setting with a dataset of 50 questions. Including a generated ontology in the GraphRAG pipeline leads to improvement over all criteria considered

Model	Comprehensive	Diversity	Empowered	Direct
Only KG	1/50	4/50	1/50	16/50
KG + ontology	**49/50**	**46/50**	**49/50**	**34/50**

In the SI, we include an ablation study showing that a randomly generated taxonomy achieves a 20/50 win rate in comprehensiveness, demonstrating some ability to retrieve information. In contrast, our generated taxonomy reaches a 30/50 win rate, highlighting that semantically correct information provides an advantage in the retrieval process.

#### 3.2.3 Expert evaluation

To assess the quality of the generated ontology, a panel of two experts conducted an evaluation of the taxonomical relationships. The experts randomly sampled relationships from various iterations of the ontology. Their task was to determine whether each sampled relationship was correct within the context of the domain. The results of this evaluation showed that, on average, 64.5% of the examined relationships were deemed correct by the experts. While this indicates a majority of accurate relationships, it also suggests room for improvement in the ontology generation process. Upon analysis of the incorrect relationships, the experts did not identify any clear patterns of errors. However, they provided three key observations for potential enhancement:

• Specificity: the quality of relationships could be improved by making them more specific, thereby reducing ambiguity and increasing precision.

• Context dependency: some extracted vocabulary terms are heavily context-dependent and may lose their intended meaning when placed in a more general context. This suggests a need for better context preservation or clearer domain boundaries.

• Semantic redundancy: the taxonomy contains instances of semantically similar concepts repeated in different parts of the structure. This redundancy could be addressed to streamline the ontology.

These expert insights provide valuable direction for refining the ontology generation process and improving the overall quality of the taxonomical relationships.

To provide a qualitative perspective on this evaluation, we include in the SI several fragments of different subtrees, such as Single-Atom Catalysts, Catalytic Performance, Characterization, and Support Materials. These examples show that, while the generated taxonomy sometimes produces relationships that may depend on context (*e.g.*, Matrix isA Support Material), and in some cases the hierarchy could be refined further (*e.g.*, Electrocatalytic Activity isA Catalytic Performance and Activity isA Catalytic Performance, rather than Electrocatalytic Activity isA Activity), the taxonomy overall successfully identifies and organizes topics within the appropriate categories, considerably reducing the amount of manual labour that would require otherwise.

## 4 Conclusion

Extraction of structured knowledge from scientific literature is a challenging task that is often performed manually by domain experts. Given the success of LLMs in NLP tasks, this work proposes the use of LLMs as information extraction engines from the scientific literature. A five-step pipeline is proposed that is able to generate ontologies and KGs with an in-context nature and without the need for any training data. The pipeline is then implemented entirely using openly available LLMs and long-context LLMs. The evaluation of the approach is done by reconstructing an existing KG and ontology of chemical elements and functional groups from the literature. The results show good accuracy in reconstructing the taxonomies and KGs with a term accuracy and hierarchical accuracy of over 80% in all iterations, and knowledge graph accuracy of over 70% in all iterations. It was observed, however, that the quality of the reconstruction is dependent on the first iteration generation. Additionally, given the stochastic nature of the LLMs, there is variability in the results. The method was then applied to the emerging domain of SACs. Given the recent development of the field, the literature lacks structured information, which makes the generation of an ontology and KG a challenging task. It was shown that the in-context generated ontology of SACs was able to improve the performance of the GraphRAG pipeline in question-answering tasks related to SACs. Finally, the quality was evaluated by a group of experts, who evaluated that 64.5% of the relationships were correct. We can thus conclude that the proposed approach is able to extract meaningful information from scientific literature.

## Author contributions

Conceptualization – MH, AMB. Data curation – AO, ML. Experiments – AO. Supervision – PS, MH, AMB. Writing: original draft – AO, PS. Writing: review & editing – all authors.

## Conflicts of interest

There are no conflicts to declare.

## Supplementary Material

DD-005-D5DD00275C-s001

## Data Availability

All the code and data used to extract the ontologies and perform the evaluations described in this work are available *via* GitHub at https://github.com/schwallergroup/ontorag/tree/main/src/OntoGen and *via* Zenodo at https://doi.org/10.5281/zenodo.17968980.^64^ Supplementary information (SI): the technical implementation and hyperparameters of the OntoGen pipeline, self-consistency methodology, prompt example, qualitative and failure-case analyses, and ablation studies validating key design choices. See DOI: https://doi.org/10.1039/d5dd00275c.
