# Supplementary material for: Scientific knowledge graph and ontology generation using open large language models
Source: Digit Discov. 2026 Feb 16;5(3):1269–79. doi: 10.1039/d5dd00275c (PMC12928120; doi:10.1039/d5dd00275c)
Supplement: DD-005-D5DD00275C-s001 [file DD-005-D5DD00275C-s001.pdf]

Supplementary Information (SI) for Digital Discovery.
This journal is © The Royal Society of Chemistry 2026

Scientific Knowledge Graph and Ontology Generation using Open Large Language Models

Alexandru Oarga,[abc] Matthew Hart,[abd] Andres M Bran,[ab‡] Magdalena Lederbauer,[abe] and Philippe Schwaller[a‡]

A Technical Stack and Experimental Setting

The following technical stack was used for the pipeline implementation:

- *Plain text extraction*: Plain text was extracted from the paper's PDF files using Nougat[1] with model `0.1.0-base`. When the extraction with Nougat failed, extraction resorted to PyMuPDF [1].
- *Terms extraction*: The split of the initial text into sentences is done with a RoBeRTa base model[2] from the Spacy suite [2]. Multiple sentences are then concatenated as long as they don't exceed a maximum length of 2048 characters. Vocabulary extraction was performed with a Llama 3.1 70B model[3] quantized to 4 bits, with a maximum context length of 1024 tokens. It was observed that a greedy generation (temperature=0) provided the best results. For the other parameters, we set $k = 5$.
- *Category generation*: The *generation* step is performed with Llama-3 8B Gradient Instruct 1048k quantized to 8 bits, context length limit of 128K tokens and temperature=0.7. The *refinement* step was done with Llama-3.1 70B quantized to 4 bits, context limit of 32K tokens and temperature=0.5. After each of the two steps, an extra prompt is performed with the former model to format the output of the LLMs. A total of 80 different samples were generated both in the generation and refinement steps.
- *Taxonomy generation and relationships extraction*: Both taxonomy generation and relationships extraction are done with a Llama 3.1 70B model[3] quantized to 4 bits, with a maximum context length of 32K tokens and temperature=0.1. For each LLM query, self-consistency was applied by taking the majority voting of 3 sampled answers.

For the GraphRAG setting, the following hyperparameters were used:

- *Text split and vectorization:* Chunk length of 1200 characters with a 100 characters overlap. Text embeddings were computed using the `all-MiniLM-L6-v2` pretrained model from Sentence Trasnformers framework[4].
- *Node2vec[5]:* Dimensionality of 1536, with 10 walks of length 40 and window size 2.

[1] `github.com/pymupdf/PyMuPDF`
[2] `github.com/explosion/spaCy`

- *Clustering:* Clustering was performed with the Leiden algorithm[6] as in the original GraphRAG work. Maximum graph cluster size of 10 nodes.
- *LLM evaluation:* The LLM evaluation of the answers was done using `claude-3-5-sonnet-20240620`.

LLM inference was done using the Ollama suite [3]. All the technical decisions made were adjusted to a budget of a single NVIDIA RTX A6000 graphics unit with 48GB. In Table 1, we include the wall clock time required to generate the reconstructed taxonomy for the ElementKG experiment in this setting. The source code is publicly available under MIT Licence.

**Method**	**Wall Clock Time (s)**
OLfT	499.63
LLM4OL	39732.20
OntoGen (Ours)	5025.47

Table 1: Wall clock time comparison of different methods for taxonomy reconstruction in the elements and functional group setting. For OntoGen, we report a single iteration time.

B Self-Consistency

The improvement of LLMs' capabilities to generate high-quality, hallucination-free answers is currently a highly active area of research. Many generic methods have been proposed that improve LLMs outputs without training data, fine-tuning or reinforcement learning, which includes, among others, self-consistency[7], debating LLMs[8], and self-refinement[9]. Research by Huang et al.[10] demonstrates that self-consistency offers competitive results while being more computationally efficient compared to other methods. Therefore, in this work, self-consistency is used to improve the quality of answers from a LLM. As utilized in our approach, self-consistency can be defined as:

Definition B.1 *Let $a_1, a_2, ..., a_n \in \mathbb{A}$ be the answers to a given prompt p generated by a LLM, and r_i the set of tokens generated before the answer a_i.*

Self-Consistency *(SC) applies a marginalization over r_i by taking the majority vote of the answers a_i, i.e. $a = \arg\max_{a_i} \sum_{j=1}^{n} \mathbb{1}(a_i = a_j)$, thus giving as a final answer the most "consistent" answer generated by the LLM.*

It is important to note that self-consistency was initially proposed to enhance Chain of Thought (CoT) reasoning[11] in LLMs[7], to improve performance on generalized problem-solving tasks. In our work, we leverage the generalizability of self-consistency to improve the quality of our knowledge schemas reconstruction.

[3] `github.com/ollama/ollama`

C Prompt Sample

This Section includes a sample question and answer obtained from the taxonomy generation phase. Note that in the question below, given the restriction in the context length, the whole taxonomy cannot be included in the prompt. As a consequence, only the term and the main category it belongs (in parenthesis) are included. Notice also that, in the answer provided, not all the terms as classified, as some of them are classified as 'None'.

Sample Question

Given this context:
===
The actinide () or actinoid () series encompasses at least the 14 metallic chemical elements in the 5f series, with atomic numbers from 89 to 102, actinium through nobelium. (Number 103, lawrencium, is sometimes also included despite being part of the 6d transition series.) The actinide series derives its name from the first element in the series, actinium. The informal chemical symbol An is used in general discussions of actinide chemistry to refer to any actinide.
All the actinides are f-block elements. Lawrencium is sometimes considered one as well, despite being a d-block element and a transition metal. The series mostly corresponds to the filling of the 5f electron shell, although as isolated atoms in the ground state many have anomalous configurations involving the filling of the 6d shell due to interelectronic repulsion. In comparison with the lanthanides, also mostly f-block elements, the actinides show much more variable valence. They all have very large atomic and ionic radii and exhibit an unusually large range of physical properties. While actinium and the late actinides (from curium onwards) behave similarly to the lanthanides, the elements thorium, protactinium, and uranium are much more similar to transition metals in their chemistry, with neptunium, plutonium, and americium occupying an intermediate position.
All actinides are radioactive and release energy upon radioactive decay; naturally occurring uranium and thorium, and synthetically produced plutonium are the most abundant actinides on Earth. These have been used in nuclear reactors, and uranium and plutonium are critical elements of nuclear weapons. Uranium and thorium also have diverse current or historical uses, and americium is used in the ionization chambers of most modern smoke detectors. Of the actinides, primordial thorium and uranium occur naturally in substantial quantities.
===
,and given the following taxonomy:
===
Carbon (Element of the periodic table)
Element of the periodic table
Hemiacetals (Functional group)
R2C=O (Functional group)
Aldehyde hydrate (Functional group)
Carbonyl (Functional group)
Aldehyde (Functional group)
Mixed acetal (Functional group)
Ketone hydrate (Functional group)
Carbonyl compounds (Functional group)
Acetal (Functional group)
Esters (Functional group)
Ketone-derived structures (Functional group)
Symmetric acetal (Functional group)
Orthoester (Functional group)
Hydroxyl group (Functional group)
Ketal (Functional group)
Functional group
===

Complete the following list to classify the terms into the taxonomy according to the context.
If a term does not fit in any of the categories, say "None".
If the text is not clear enough to classify a term, say "None".
If the text does not explicitly mention that a term is a type of another, say "None".
If multiple categories apply, choose the most specific one.
In the ouput include only the classification. Do not include any explanation or additional information.
Do not classify a term to be its own parent, this is, do not output answers such as "A isA A".
The answer should include 'isA'.

Nuclear weapons isA
Lawrencium isA
F-block elements isA
Thorium isA
Radioactive decay isA
Actinides isA
Actinoid isA
Neptunium isA
Actinium isA
Actinide isA
Curium isA
Ground state isA
Primordial thorium isA
Americium isA
6d transition series isA
Plutonium isA
Protactinium isA
Lanthanides isA
Atomic numbers isA
Smoke detectors isA
Nuclear reactors isA
Ionic radii isA
Uranium isA
Electron shell isA
Transition metals isA
D-block element isA
Ionization chambers isA
5f series isA
Valence isA
Interelectronic repulsion isA
Nobelium isA
Metallic chemical elements isA

Sample Answer

Here is the list of classifications:

- Nuclear weapons isA None
- Lawrencium isA Actinide
- F-block elements isA None
- Thorium isA Actinide
- Radioactive decay isA None
- Actinides isA Metallic chemical elements
- Actinoid isA Actinide
- Neptunium isA Actinide
- Actinium isA Actinide
- Actinide isA F-block elements
- Curium isA Actinide
- Ground state isA None
- Primordial thorium isA Thorium
- Americium isA Actinide
- 6d transition series isA Transition metal
- Plutonium isA Actinide
- Protactinium isA Actinide
- Lanthanides isA F-block elements
- Atomic numbers isA None
- Smoke detectors isA None
- Nuclear reactors isA None
- Ionic radii isA None
- Uranium isA Actinide
- Electron shell isA None
- Transition metals isA Metallic chemical elements
- D-block element isA Transition metal
- Ionization chambers isA None
- 5f series isA F-block elements
- Valence isA None
- Interelectronic repulsion isA None
- Nobelium isA Actinide
- Metallic chemical elements isA Element of the periodic table

Property	ElementKG	Iteration 1	Iteration 2	Iteration 3	Iteration 4
#nodes	272	558.6 ± 74.4	668.8 ± 60.1	720.0 ± 63.1	762.0 ± 58.4
#isA	347	591.8 ± 81.5	764.0 ± 72.1	839.8 ± 73.9	922.0 ± 48.3
#leaves	188	509.4 ± 80.6	602.4 ± 70.6	646.0 ± 75.4	681.6 ± 70.7
Depth	8	6.2 ± 1.0	6.6 ± 0.8	7.0 ± 0.6	7.2 ± 1.0
Max #isA per node	32	213.2 ± 40.7	264.8 ± 11.3	275.4 ± 13.5	286.2 ± 15.9
Mean #isA per node	1.28	1.06 ± 0.02	1.14 ± 0.05	1.17 ± 0.06	1.21 ± 0.06
Mean steps to leaf	3.63	2.78 ± 0.51	2.80 ± 0.54	2.86 ± 0.52	2.89 ± 0.51

Table 2: Taxonomical properties of the reconstructed ElementKG across iterations averaged over five runs.

Property	Iteration 1	Iteration 2	Iteration 3	Iteration 4	Iteration 5
#nodes	1188	1344	1368	1426	1426
#isA	1239	1520	1603	1832	1845
#leaves	1151	1302	1317	1370	1370
Mean #isA per node	1.0429	1.1310	1.1718	1.2847	1.2938
Max #isA per node	208	226	229	240	240
Depth	5	6	9	9	9
Mean steps to leaf	2.3594	2.3832	2.3911	2.3822	2.3822

Table 3: Taxonomical properties of the generated SACs taxonomy across iterations.

D Taxonomical Properties

This Sections includes details of the taxonomical properties obtained with the two taxonomies reconstructed in this work. Table 2 includes the resulting taxonomical properties obtained for ElementKG reconstruction. Note that the original ElementKG is considerably smaller in comparison, as this taxonomy has been narrowed down to only the required terms, while this work reconstructs a general-purpose taxonomy. Table 3 presents the main taxonomical properties of the SACs taxonomy.

E Additional Results

E.1 Chemical Elements and Functional Groups Reconstruction

E.1.1 Failure Cases

This Section includes four examples with representative failure cases from the ElementKG reconstruction setting. In Example 1, the functional group tree is incomplete because the class *Groups Containing Sulfur* is missing. Example 2 shows a similar issue in the elements subtree, where the intermediate classes *Non-Ferrous Metals* and *Non-Ferrous Light Metals* are omitted. In Example 3 we can see that *Hg* is classified under *Non Ferrous Metals*, *Non Ferrous Heavy Metals* in ground truth and under *Transition Metals*, *Post Transition Metals* in the reconstructed hierarchy. While both classifications are correct, they differ from one another. Finally, in Example 4, *Sulfhydryl* should be represented as a subclass of *Thiol*, but in the reconstructed version, both appear at the same level under *Functional Group*.

Example 1.

Ground Truth

```
Functional Group
|- Groups Containing Sulfur
   |- Disulfide
   |- Sulfide
   |- Thial
   |- Thioketone
   |- Thiol
   |- <omitted>
```

Reconstructed

```
Functional Group
|- Disulfide
|- Sulfide
|- Thial
|- Thioketone
|- Thiol
|- <omitted>
```

Example 2.

Ground Truth

```
Element
|- Metals
   |- Non Ferrous Metals
      |- Non Ferrous Light Metals
         |- Na
         |- Al
         |- Ca
         |- <omitted>
```

Reconstructed

```
Element
|- Metals
   |- Na
   |- Al
   |- Ca
   |- <omitted>
```

Example 3.

Ground Truth

```
Element
|- Metals
   |- Non Ferrous Metals
      |- Non Ferrous Heavy Metals
         |- Hg
```

Reconstructed

```
Element
|- Metals
   |- Transition Metals
      |- Post Transition Metals
         |- Hg
```

Example 4.

Ground Truth

```
Functional Group
|- Thiol
  |- Sulfhydryl
```

Reconstructed

```
Functional Group
|- Thiol
|- Sulfhydryl
```

E.2 Ontology and KG Generation for Single Atom Catalysis

E.2.1 Qualitative Results

This section presents four examples with subtrees from the Single-Atom Catalysis taxonomy.

- Single-Atom Catalysts Subtree: The tree correctly includes different types of SACs such as *Pt1/FeOx*, *Ir1/CeO2* or *Pt1/m Al2O3*. In general, the terms under *Single-Atom Catalysts* are accurate, although the hierarchy could be refined. For instance, *Ni SA/ZrO2* could be classified as a subtype of *Nickel single-atom catalyst*.

- Catalytic Performance Subtree: This subtree appropriately classifies common catalytic performance properties such as *Selectivity*, *Current Density*, and *Faradaic Efficiency*. It also includes certain terms that might appear out of place without additional context, such as *h@1* or *FF*.

- Characterization Subtree: effectively captures a diverse range of characterization methods used for SACs, such as *In situ spectroscopy*, *Operando X ray absorption near edge structure spectroscopy* and *Extended X-ray absorption fine structure*.

- Support Materials Subtree: The subtree includes materials commonly used as supports for SACs, such as *Ag (100)*, *N-doped carbon matrix*, or *Aluminia*. It also contains terms that may seem unclear without further context, such as *Matrix*.

SACs Subtree

```
Thing
|- Material
   |- Catalyst
      |- Single atom catalysts
         |- Single atomic Rh catalyst
         |- Single-atom electrocatalysts
         |- Single atom form
         |- Pt1/m Al2O3
         |- Ir1/CeO2
         |- Pt single atoms
         |- Nickel single-atom catalyst
         |- Model SACs
         |- M1@Co/NC catalysts
         |- Ir2@Co/NC catalyst
         |- Single atom Pt catalyst
         |- Ni SA /ZrO2
         |- metal single atom catalysts
         |- Silver single atom catalysts
         |- Single atom Ag on ATO catalyst
         |- Noble metal single-atom catalysts
         |- Pt1/CeOx TiO2
         |- Pt1/q-Al2O3
         |- Metallic SACs
         |- Pt1/a-MoC
         |- Pt SACs
         |- Pt1/FeOx
         |- Pt1/TiO2
         |- <omitted for clarity>
```

Catalytic Peformance Subtree

```
Thing
|- Catalytic performance
   |- Current density
   |- selectivity
   |- CO Faradic efficiency
   |- Selectivity issues
   |- Energy inefficient
   |- Electrocatalytic properties
   |- mA cm-2
   |- Catalyst performance
   |- Catalytic properties
   |- Mass activity
   |- Activity
   |- Stabilities
   |- Durability
   |- FF
   |- Performance
   |- Low-temperature activity
   |- Specific activity
   |- Electrocatalytic activity
   |- Chemoselectivity
   |- Catalyst activity
   |- Faradaic efficiency (FE)
   |- Turnover frequency (TOF)
   |- h@1
   |- CO selectivity
   |- Product selectivity
   |- Photocatalyst activity
   |- CO2 photoreduction activity
   |- <omitted for clarity>
```

Charaterization Subtree

```
Thing
|- Characterization
   |- Characterization Tools
      |- In situ DRIFTS
      |- Experimental observation
      |- Electron paramagnetic resonance
      |- In situ spectroscopy
      |- Diffuse reflectance infrared Fourier transform spectroscopy (DRIFTS)
      |- Operando X ray absorption near edge structure spectroscopy
      |- Near ambient X ray photoelectron spectroscopy
      |- Raman spectroscopy
      |- Extended X-ray absorption fine structure (EXAFS) analysis
      |- X ray absorption near edge spectroscopy
      |- X-ray diffraction
      |- Operando spectroscopy characterization
      |- X ray absorption near edge structure spectroscopy
      |- Physical characterization
      |- Electrochemical measurements
      |- Experimental techniques
      |- XRD pattern
      |- X-ray Photoelectron Spectroscopy (XPS)
      |- Optical Emission Spectroscopy
      |- FT
      |- X-ray absorption near-edge structure
      |- ICP OES
      |- SAED
      |- 1H NMR spectroscopy
      |- Fourier transform
      |- X-ray absorption fine structure spectra
      |- X ray adsorption fine structure analysis
      |- HAADF-STEM
      |- Aberration-corrected high-angle annular dark-field scanning transmission electron
microscopy
      |- High resolution TEM
      |- TPD data
      |- TPD IR
      |- Fourier Transform Infrared (FTIR)
      |- Scanning Transmission Electron Microscopy
      |- <omitted for clarity>
```

Support Materials Subtree

```
Thing
|- Support
   |- Support Materials
      |- Oxide supports
      |- Au (100)
      |- Defective graphene
      |- Ag (100)
      |- Conductive carbon support
      |- Heterogeneous support
      |- Carbon matrix
      |- Co/NC support
      |- Defect rich zirconia
      |- Antimony-doped tin oxide (ATO)
      |- Tin oxide
      |- ATO support
      |- Conductive support
      |- Aqueous electrolytes
      |- N doped carbon matrix
      |- Alumina support
      |- Sb SnO2
      |- S carbon
      |- Carbon support
      |- Stoichiometric surface
      |- Matrix
      |- <omitted for clarity>
```

E.2.2 Manual Curation

This section details the manual curation process used to generate the main categories of the SACs taxonomy. Starting from the 121 categories listed on the left, we manually grouped them into 12 broader categories shown on the right. It is important to note that this grouping is not unique and that multiple valid alternatives exist. The final choice ultimately depends on the specific downstream application for which the ontology is intended.

Clustering of Main Categories

Catalytic Reactions<br>Carbon Monoxide Reduction Reaction<br>Electrochemical CO2 Reduction Reaction (CO2RR)<br>Electrochemical Nitrogen Reduction Reaction (NRR)<br>Electrochemical Reaction<br>Electrochemical Reactions and Electrocatalysts<br>Heterogeneous CO2 Reduction Reaction (CO2RR)<br>OER (Oxygen Evolution Reaction)<br>Other Reactions<br>Other reactions<br>Oxygen Reduction Reaction (ORR)<br>Reaction Kinetics<br>Reaction and Mechanism<br>Reaction and Synthesis<br>Photocatalysis<br>Energy Conversion	Reactions
Support<br>Support Materials<br>Supports	Supports
Characterization Techniques<br>Characterization of the Catalysts<br>Characterization of the Catalysts (or Materials)<br>Synthesis and Characterization<br>Spectroscopy	Characterization
Properties<br>Properties and Kinetic Analysis<br>Properties of Catalysts<br>Properties of the Catalyst<br>Catalyst Properties<br>Catalyst Structure and Properties<br>Chemical Properties<br>Electrochemical Properties<br>Synthesis and Properties<br>Structure/Properties<br>Adsorption and Dissociation<br>Coordination Environment<br>Crystal<br>Kinetic analysis	Properties
Structure & Synthesis<br>Synthesis<br>Synthesis Methods<br>Synthesis Strategies<br>Catalyst Design<br>Electrodeposition Method	Synthesis
Applications<br>Applications for Other Reactions<br>Applications and Materials<br>Applications and Stability<br>Applications for Water-Splitting or Related Energy Conversion<br>Applications in Energy Conversion and Storage<br>Catalyst Performance and Applications<br>Catalyst Performance and Applications of SAHCs<br>Catalytic Performance and Applications<br>Electrochemical Applications<br>Materials and Applications<br>Other Applications of Single-Atom Catalysts (SACs)	Applications

Catalytic Mechanism<br>Mechanism<br>Mechanism of Catalysis<br>Mechanism of Catalytic Process<br>Mechanism of Electron Transfer<br>Mechanism of Photocatalytic Process<br>Mechanism of the Catalytic Process<br>Mechanism of the Photocatalytic Process<br>Mechanism of the Reaction<br>Mechanism of the catalytic process<br>Mechanism of the photocatalytic process<br>Mechanisms	Mechanisms
Materials Preparation and Engineering<br>Preparation<br>Preparation Methods	Preparation
Theoretical Methods<br>Theoretical Models<br>Theoretical Studies<br>Theoretical Study<br>Theoretical Study on Mechanism and Activity/Selectivity<br>Theoretical Study on the Mechanism and Activity/Selectivity<br>Theoretical and Computational<br>DFT Studies on the Nature of Active Sites<br>DFT calculation<br>Theoretical Studies for MOFs-Template Synthesis of SAHCs	Theory and Modelling
Catalysts with Different Structures and Composition<br>Electron Structure<br>Electron structure<br>Nanostructure and Morphology<br>Nanostructured Transition Metal Clusters<br>Cation<br>Ligand<br>Morphology<br>Surface<br>Surface Sites<br>Transition Metal-Element	Structures
Carbon Materials<br>Functional Materials<br>Materials<br>Materials Science<br>Materials and Chemical Engineering<br>Nanomaterials<br>Catalysts<br>Electrochemical Catalysis<br>Heterogeneous Catalysts<br>Single-Atom Catalysts (SACs)<br>Substrate	Materials
Catalyst Performance Evaluation<br>Catalyst Performance Evaluation Method<br>Catalytic Performance<br>Catalytic Performance Evaluation<br>Stability<br>Activity and Selectivity	Performance

Category	Metric	OntoGen (w/o SC)	OntoGen
All	Hierarchical Precision (↑)	0.742 ± 0.053	$\mathbf{0.836 \pm 0.038}$
	Hierarchical Recall (↑)	$\mathbf{0.679 \pm 0.045}$	0.660 ± 0.024
	Hierarchical F1 (↑)	0.708 ± 0.040	$\mathbf{0.737 \pm 0.023}$
	Hierarchical Accuracy (↑)	$\mathbf{0.931 \pm 0.005}$	0.924 ± 0.011
Element	Hierarchical Precision (↑)	0.674 ± 0.052	$\mathbf{0.839 \pm 0.057}$
	Hierarchical Recall (↑)	$\mathbf{0.631 \pm 0.096}$	0.613 ± 0.056
	Hierarchical F1 (↑)	0.646 ± 0.049	$\mathbf{0.707 \pm 0.049}$
	Hierarchical Accuracy (↑)	$\mathbf{0.959 \pm 0.005}$	0.951 ± 0.018
Functional Group	Hierarchical Precision (↑)	0.727 ± 0.146	$\mathbf{0.765 \pm 0.132}$
	Hierarchical Recall (↑)	$\mathbf{0.595 \pm 0.024}$	0.569 ± 0.029
	Hierarchical F1 (↑)	0.645 ± 0.068	$\mathbf{0.647 \pm 0.061}$
	Hierarchical Accuracy (↑)	$\mathbf{0.894 \pm 0.007}$	0.888 ± 0.010

Table 4: Ablation of self-consistency in taxonomy generation. We ablate the application of a self-consistency step for each of the queries performed in the taxonomy generation pipeline. Overall, including self-consistency improves the quality of the reconstructed taxonomy. Best values are highlighted in bold.

F Ablation Study

F.1 Chemical Elements and Functional Groups Reconstruction

In this Section, we ablate the taxonomy generation procedure in the element and functional group reconstruction setting. In Table 4 we ablate the use of self-consistency (SC) in the procedure. The results show that without SC, more relationships are included in the taxonomy, which slightly increases hierarchical recall from 0.66 (with SC) to 0.67 (without SC). However, this also leads to the inclusion of many incorrect relationships, significantly lowering precision from 0.83 (with SC) to 0.74 (without SC). Contrarily, applying SC raises precision while maintaining competitive recall. As a result, the overall F1-score is higher with SC (0.73 vs. 0.70), indicating that self-consistency improves the overall quality of the reconstruction.

F.2 Ontology and KG Generation for Single Atom Catalysis

F.2.1 Vocabulary Extraction

In this Section, we ablate key steps of our proposed approach and evaluate it in the SACs vocabulary setting presented in the paper. In Table 5 we ablate our vocabulary extraction process. Particularly, we analyze the effects of (i) splitting the text into sentences before extracting terms, and (ii) applying a verification step that discards hallucinated terms (i.e., terms extracted by the LLM that are not present in the original text).

From this table, we can see that when the text is not split into sentences,

	Ours (No split)	Ours (No verif.)	Ours
**Exact match**			
Precision (↑)	$\mathbf{0.691 \pm 0.154}$	0.595 ± 0.158	0.671 ± 0.150
Recall (↑)	0.435 ± 0.105	0.439 ± 0.097	$\mathbf{0.483 \pm 0.092}$
F1 (↑)	0.529 ± 0.120	0.501 ± 0.116	$\mathbf{0.558 \pm 0.112}$
# Gold Terms	43.0 ± 15.4	43.0 ± 15.4	43.0 ± 15.4
# Predictions	26.4 ± 6.6	31.5 ± 7.5	31.6 ± 11.9
**Substring**			
Precision (↑)	$\mathbf{0.935 \pm 0.090}$	0.852 ± 0.108	0.909 ± 0.098
Recall (↑)	0.595 ± 0.095	0.642 ± 0.073	$\mathbf{0.667 \pm 0.083}$
F1 (↑)	0.719 ± 0.082	0.725 ± 0.055	$\mathbf{0.762 \pm 0.064}$
# Gold Terms	43.0 ± 15.4	43.0 ± 15.4	43.0 ± 15.4
# Predictions	26.4 ± 6.6	31.5 ± 7.5	31.6 ± 11.9
**Word**			
Precision (↑)	0.945 ± 0.058	0.860 ± 0.088	$\mathbf{0.947 \pm 0.058}$
Recall (↑)	0.640 ± 0.121	0.707 ± 0.088	$\mathbf{0.686 \pm 0.103}$
F1 (↑)	0.756 ± 0.095	0.772 ± 0.067	$\mathbf{0.790 \pm 0.075}$
# Gold Terms	94.7 ± 25.9	94.7 ± 25.9	94.7 ± 25.9
# Predictions	62.1 ± 13.3	76.5 ± 16.5	68.0 ± 18.4

Table 5: Ablation of vocabulary extraction in the SACs domain. We ablate splitting the text into sentences before extraction and performing a verification step to remove hallucinated terms. Overall, removing each of the steps leads to a decrease in the quality of the extracted vocabulary. Best values are highlighted in bold.

the number of extracted terms is lower on average. It can be seen that, as a consequence, the exact match and substring match precision are higher than if we split the text, with 0.69 and 0.67, respectively. As a result, the exact-match and substring-match precision values are higher (0.69 and 0.67, respectively). However, the smaller set of extracted terms reduces recall (0.43 and 0.59 without splitting, compared to 0.48 and 0.66 with splitting). As a consequence, the F1-scores are consistently higher when sentence splitting is applied: 0.55 vs. 0.52 for exact match, 0.76 vs. 0.71 for substring match, and 0.79 vs. 0.75 for single-word match.

Similarly, in the table, we also ablate the verification step, where we discard hallucinated terms. As expected, including potentially hallucinated terms reduces performance, with F1-scores of 0.50 (exact match), 0.72 (substring match), and 0.77 (single-word match). On the other hand, when the verification step is applied, all metrics improve to 0.55, 0.76, and 0.79, respectively. This demonstrates the benefit of this verification step for the overall performance.

F.2.2 GraphRAG-Based Downstream Evaluation

In this Section, we ablate the GraphRAG-based downstream application results presented in Section 3.2.1 of the paper. To evaluate the utility of the generated ontology for downstream tasks, we re-evaluate the GraphRAG results in Table 3

Model	Comprehensive	Diversity	Empowered	Direct
KG + Random Taxonomy	20/50	20/50	18/50	22/50
KG + Taxonomy	**30/50**	**30/50**	**32/50**	**28/50**

Table 6: GraphRAG ablation for SACs domain. We ablate the taxonomy used in the GraphRAG pipeline by using a randomly generated taxonomy. We report the win-rate according to different criteria in the GraphRAG setting with a dataset of 50 questions. Using the generated ontology over a random one improves over all criteria considered.

of the paper using a randomly constructed taxonomy. This random taxonomy is generated by shuffling the node labels of the original taxonomy, therefore preserving its degree distribution while changing its semantic information. This procedure does not affect the knowledge graph edge,s which remain unchanged during this process.

In Table 6 we include the comparison on the question-answer benchmark. Regarding the comprehensiveness criteria, the random taxonomy shows some capacity to retrieve relevant context with a win rate of 20/50. However, the generated ontology clearly outperforms it with a win rate of 30/50. The same trend is observed in the other three evaluation criteria considered.

References

[1] L. Blecher, G. Cucurull, T. Scialom and R. Stojnic, *arXiv preprint arXiv:2308.13418*, 2023.

[2] Y. Liu, *arXiv preprint arXiv:1907.11692*, 2019.

[3] A. Dubey, A. Jauhri, A. Pandey, A. Kadian, A. Al-Dahle, A. Letman, A. Mathur, A. Schelten, A. Yang, A. Fan *et al.*, *arXiv preprint arXiv:2407.21783*, 2024.

[4] N. Reimers and I. Gurevych, Proceedings of the 2019 Conference on Empirical Methods in Natural Language Processing, 2019.

[5] A. Grover and J. Leskovec, Proceedings of the 22nd ACM SIGKDD international conference on Knowledge discovery and data mining, 2016, pp. 855–864.

[6] V. A. Traag, L. Waltman and N. J. Van Eck, *Scientific reports*, 2019, **9**, 1–12.

[7] X. Wang, J. Wei, D. Schuurmans, Q. Le, E. Chi, S. Narang, A. Chowdhery and D. Zhou, *arXiv preprint arXiv:2203.11171*, 2022.

[8] Y. Du, S. Li, A. Torralba, J. B. Tenenbaum and I. Mordatch, *arXiv preprint arXiv:2305.14325*, 2023.

[9] A. Madaan, N. Tandon, P. Gupta, S. Hallinan, L. Gao, S. Wiegreffe, U. Alon, N. Dziri, S. Prabhumoye, Y. Yang *et al.*, *Advances in Neural Information Processing Systems*, 2024, **36**, year.

[10] J. Huang, X. Chen, S. Mishra, H. S. Zheng, A. W. Yu, X. Song and D. Zhou, *arXiv preprint arXiv:2310.01798*, 2023.

[11] J. Wei, X. Wang, D. Schuurmans, M. Bosma, F. Xia, E. Chi, Q. V. Le, D. Zhou *et al.*, *Advances in neural information processing systems*, 2022, **35**, 24824–24837.
